# Radioembolisation with yttrium‒90 microspheres versus sorafenib for treatment of advanced hepatocellular carcinoma (SARAH): study protocol for a randomised controlled trial

**DOI:** 10.1186/1745-6215-15-474

**Published:** 2014-12-03

**Authors:** Valérie Vilgrain, Mohamed Abdel-Rehim, Annie Sibert, Maxime Ronot, Rachida Lebtahi, Laurent Castéra, Gilles Chatellier

**Affiliations:** Department of Radiology, Assistance Publique, Hôpitaux de Paris, APHP, University Hospitals Paris Nord Val de Seine, Beaujon, 100 boulevard General Leclerc 92118 Clichy Hauts-de-Seine, France; University Paris Diderot, Sorbonne Paris Cité, Paris, France, INSERM U1149, Centre de Recherche de l’Inflammation (CRI), 16 rue Henri Huchard, 75018 Paris, France; Department of Nuclear Medicine, Assistance Publique, Hôpitaux de Paris, APHP, University Hospitals Paris Nord Val de Seine, Beaujon, 100 boulevard General Leclerc, 92118 Clichy Hauts-de-Seine, France; Department of Hepatology, Assistance Publique, Hôpitaux de Paris, APHP, University Hospitals Paris Nord Val de Seine, Beaujon, 100 boulevard General Leclerc, 92118 Clichy Hauts-de-Seine, France; University Paris Diderot, Sorbonne Paris Cité, 5 rue Thomas Mann, 75013 Paris, France; Assistance Publique, Hôpitaux de Paris, APHP, Hôpital Européen Georges Pompidou, URC, 20 rue Leblanc, 75015 Paris, France; INSERM, Centre d’Investigation Épidémiologique 4 (CIE4), 80 rue Lecourbe, 75015 Paris, France; Sorbonne Paris Cité, Faculté de Médecine, Université Paris-Descartes, 12 rue de l'Ecole de Médecine, 75006 Paris, France; the SARAH trial group, France

**Keywords:** Advanced hepatocellular carcinoma, Radioembolisation (RE), SIR-Spheres™ microspheres, Sorafenib

## Abstract

**Background:**

Untreated advanced hepatocellular carcinoma (HCC) is linked to poor prognosis. While sorafenib is the current recommended treatment for advanced HCC, radioembolisation (RE; also called selective internal radiation therapy or SIRT) with yttrium-90 microspheres has shown efficacy in cohort studies. However, there are no head-to-head trials comparing radiation therapy with yttrium-90 microspheres and sorafenib in advanced HCC. The SARAH trial has been designed to compare the efficacy and safety of sorafenib therapy and RE using yttrium-90 resin microspheres (SIR-Spheres™; Sirtex Medical Limited, North Sydney, Australia) in patients with advanced HCC. Quality of life (QoL) and cost-effectiveness will also be compared between therapies.

**Methods/Design:**

SARAH is a prospective, randomised, controlled, open-label, multicentre trial comparing the efficacy of RE with sorafenib in the treatment of patients with advanced HCC. The trial aims to recruit adults with a life expectancy of >3 months, Eastern Cooperative Oncology Group (ECOG) performance status ≤1, and: advanced HCC according to the Barcelona criteria (stage C) or recurrent HCC after surgical or thermoablative treatment who are not eligible for surgical resection, liver transplantation or thermal ablation; or two rounds of failed chemoembolisation. Patients will be randomised 1:1 to receive either RE or sorafenib 400 mg twice daily. All patients will be monitored for between 12 and 48 months following start of treatment. The primary endpoint of the SARAH trial is overall survival (OS). Secondary endpoints include: adverse events, progression-free survival at 6 months; tumour response rate; general or liver disease-specific QoL scores; and cost of each treatment strategy. Assuming an increase in median OS of 4 months with RE versus sorafenib therapy, randomising at least 400 patients (200 in each treatment arm) will be sufficient for 80% power and a bilateral alpha risk of 5%; therefore, 440 patients will be enrolled to allow for 10% loss of patients due to ineligibility.

**Discussion:**

The SARAH trial is the first randomised head-to-head study to compare RE with sorafenib in advanced HCC, and will establish the potential role of RE in HCC treatment guidelines.

**Trial registration:**

ClinicalTrials.gov identifierNCT01482442, first received 28 November 2011

**Electronic supplementary material:**

The online version of this article (doi:10.1186/1745-6215-15-474) contains supplementary material, which is available to authorized users.

## Background

The prevalence and incidence of hepatocellular carcinoma (HCC) is highly variable in different regions of the world but the burden is predicted to increase in the coming years[1]. In developed countries, early diagnosis of HCC is possible in 30 to 60% of patients, and as a result, HCC is often diagnosed in the advanced stage of disease (stage C of the Barcelona Clinic Liver Cancer classification – that is, ECOG performance status 1 to 2, portal invasion or extrahepatic spread, and Child-Pugh A-B). Curative treatment (by surgical resection, liver transplantation or thermoablative treatment) is possible only in a limited proportion of these patients[2], and many cases of HCC progress to an advanced stage following locoregional treatment. In patients with untreated advanced HCC, the prognosis is poor, with a median survival time of approximately 5 to 7 months, although this varies depending on Child‒Pugh score[3–5].

The pivotal Sorafenib Hepatocellular carcinoma Assessment Randomized Protocol (SHARP) trial showed that sorafenib (Nexavar™, Bayer HealthCare Pharmaceuticals, Berlin, Germany) treatment significantly increased median overall survival (OS) time by approximately 3 months versus placebo (10.7 months versus 7.9 months, respectively; *P* <0.001) in patients with advanced HCC[6]. These findings were subsequently confirmed in a randomised controlled trial in the Asia-Pacific, which showed OS of 6.5 months in the sorafenib arm versus 4.2 in the placebo arm (*P* <0.014)[7]. As a result of these data, sorafenib is the current recommended first-line treatment for advanced (Barcelona stage C) HCC[2]. However, while sorafenib increased OS in the SHARP study, it did not improve median time to symptomatic progression, and was associated with an overall adverse‒effect incidence of 80%. Adverse events experienced by >5% of patients in the SHARP trial included diarrhoea (13.1%), asthenia (7.4%), hand-foot skin reaction (7.0%), and erythema or desquamation (5.4%); dose reductions and treatment interruptions due to adverse effects occurred in 26% and 44% of cases, respectively[6]. As such, there is a medical need for the study of alternative treatment options for advanced HCC.

Radioembolisation (RE; also called selective internal radiation therapy or SIRT) with SIR-Spheres™ (Sirtex Medical Limited, North Sydney, Australia), which contain the β-emitter yttrium-90, is one potential alternative treatment of advanced HCC. RE enables targeted delivery of radiation to the tumours, while the surrounding liver parenchyma is largely spared. A recent meta‒analysis showed a high response rate to yttrium‒90 RE in HCC patients[8]. Population disparity prevented assessment of OS in this meta-analysis but cohort studies of patients with HCC receiving yttrium‒90 RE report median OS between 7 and 26.3 months[9–18]. Collectively, these data suggest that the use of RE for advanced HCC warrants further investigation, and might improve median OS with fewer side effects and/or better quality of life (QoL) compared with sorafenib.

To the authors’ knowledge, no controlled, prospective trials have been published on the efficacy of RE in HCC patients. For this reason, the SorAfenib versus Radioembolisation in Advanced Hepatocellular carcinoma (SARAH) trial has been designed as a prospective, randomised, open-label, multicentre trial to compare the OS in patients with advanced HCC receiving either RE with SIR-Spheres™ or sorafenib. Secondary objectives include comparisons between the treatment arms of other efficacy parameters, the safety profile and tolerability, QoL and cost-effectiveness.

## Methods/Design

The SARAH trial will be conducted in accordance with the Declaration of Helsinki and current good clinical practice guidelines, and all participating centres have obtained the relevant ethics committee approval before patient enrolment (see Additional file1).

### Eligible population

The inclusion and exclusion criteria for the SARAH trial are summarised in Table 1. Informed consent will be obtained from each participant.

**Table 1:** **Patient eligibility criteria for SARAH trial**

Inclusion criteria	Exclusion criteria
• Written informed consent provided	• Other primary tumour except for basal cell carcinomas or superficial bladder cancers
• Aged ≥18 years	
• Histologically or cytologically confirmed diagnosis, or AASLD criteria for the diagnosis, of HCC and at least one measureable lesion on CT according to RECIST criteria	• Extrahepatic metastases except non-specific pulmonary tumours <1 cm and abdominal lymph nodes <2 cm
	• Previously treated advanced HCC (excluding chemoembolisation^*^)
• Patients not eligible for surgical resection, liver transplantation or thermoablation who have advanced HCC according to the Barcelona criteria (stage C), with or without portal invasion **OR** patients with recurrent HCC (new lesion in a different place) after surgical or thermoablative treatment who are not eligible for any other treatment; **OR** patients in whom chemoembolisation has failed after two rounds – treatment failure is defined as the absence of objective response in the treated nodule after two rounds (objective response according to the modified RECIST criteria and/or EASL criteria)	• Advanced liver disease with a Child-Pugh score > B7 or active digestive haemorrhage or encephalopathy or refractory ascites
	• Pregnant or breastfeeding women
	• Allergy to contrast agents
	• Contraindication to hepatic artery catheterisation, such as severe peripheral arterial disease precluding catheterisation
	• Mental illness or other psychological disorder affecting the informed consent
	• Patient unable or unwilling to comply with the treatment and follow-up required by the study
	• Unable to take oral medication
• ECOG performance status ≤1	
• Adequate haematological function: haemoglobin ≥9 g/100 mL, neutrophils ≥1,500/mm^3^, platelets ≥50,000/mm^3^	
• Adequate kidney function: creatinine <150 μmol/L	
• Bilirubin ≤50 μmol/l, AST or ALT ≤5 x ULN, INR ≤1.5	
• If liver cirrhosis, Child-Pugh A-B7	
• Affiliated to a social security scheme or beneficiary	

^*^Patients who have not responded to chemoembolisation but who meet the other selection criteria will be included in this study. AASLD, American Association for the Study of Liver Diseases; ALT, alanine aminotransferase; AST, aspartate transaminase; CT, computed tomography; EASL, European Association for the Study of the Liver; ECOG, Eastern Cooperative Oncology Group; HCC, hepatocellular carcinoma; INR, international normalised ratio; RECIST, response evaluation criteria in solid tumours; ULN, upper limit of normal.

### Overview of trial design

**Figure 1:**
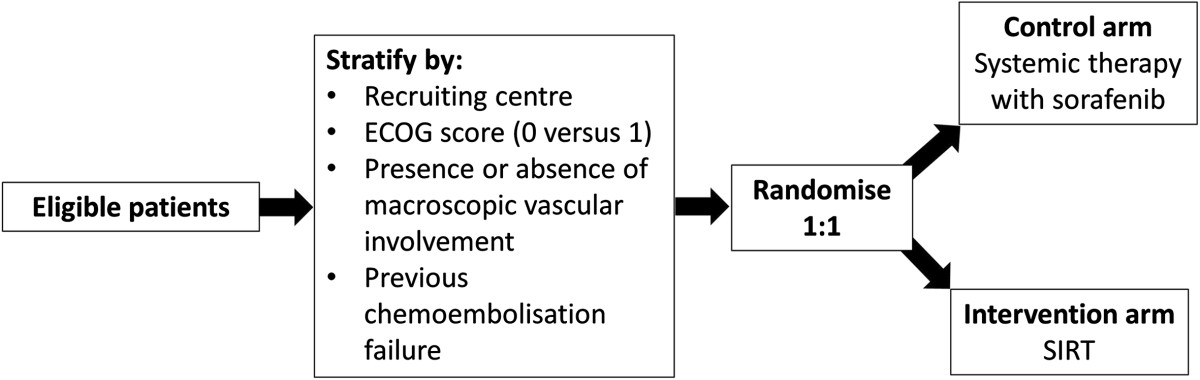
**Overview of the SARAH trial design.** ECOG, Eastern Cooperative Oncology Group; RE, radioembolisation.

SARAH is a prospective, randomised open-label, multicentre trial comparing RE and sorafenib in patients with advanced HCC. In SARAH, the aim will be to recruit a minimum of 440 patients over a period of 24 months across 28 centres in France. Centres will be chosen based on their potential to recruit a high number of patients, and the expertise in intra-arterial treatment, and will receive special training with RE. Eligible patients will be stratified 1:1 to receive either systemic therapy with oral sorafenib (control arm) or RE with SIR-Spheres™ (RE arm; Figure 1).

### Randomisation

Eligible patients will be randomised 1:1. The randomisation will be stratified by centre, Eastern Cooperative Oncology Group (ECOG) score (0 versus 1), the presence or absence of macroscopic vascular invasion seen on imaging (obstruction of the portal vein or its branches) and previous chemoembolisation failure. The list will be balanced by different sized blocks and randomly alternated. The data coordination centre will prepare the randomisation list before enrolment begins.

### Treatments

In the sorafenib arm, patients will receive oral treatment with sorafenib (400 mg twice daily) beginning in the week following randomisation (the first day of receiving treatment is defined as D0 in Table 2). Treatment suspensions and dose reductions (to 400 mg/d) will be permitted in case of adverse events (based on the SHARP study[6]) and at the treating practitioner’s discretion. Treatment may be resumed once the adverse events have been resolved, with incremental doses up to 400 mg twice daily.

**Table 2:** **SARAH trial assessment schedule**

Visits	Enrolment/randomisation	D0	D15	M1	M2	M3	M4	M5	M6	M7	M8	M9	End of participation
Identification	X												
Verification of selection criteria	X												
Consent signature	X												
Initial assessment – history	X												
CT scan	X			X		X			X			X	X
CT perfusion	X			X		X			X				
Laboratory tests	X	X	X	X	X	X	X	X	X	X	X	X	X
Classification	X			X	X	X	X	X	X	X	X	X	X
Clinical examination				X	X	X	X	X	X	X	X	X	X
Quality of life questionnaires	X			X		X			X			X	X
Preparatory angiography		X											
Scintigraphy		X											
RE		X											
Start of sorafenib treatment		X											
Retreatment^*^				X	X	X	X	X	X	X	X	X	
Cancer progression monitoring				X	X	X	X	X	X	X	X	X	X
Sorafenib monitoring				X	X	X	X	X	X	X	X	X	X
Concomitant medication				X	X	X	X	X	X	X	X	X	X
Adverse events				X	X	X	X	X	X	X	X	X	X

^*^Timing of retreatment depends upon type of retreatment (see text). CT, computed tomography; D, day; M, month; RE, radioembolisation.

In the RE arm, patients randomised to RE will require a hepatic angiogram, and a liver-to-lung shunt pre-assessment with technetium-99 m (^99m^Tc)-marked human serum albumin in order to determine their suitability for the RE procedure. Accessory tumoural vascular branches and extrahepatic vascular branches will be embolised using thrombogenic coils or vascular plugs in order to isolate the arterial supply of the afferent vessel that vascularises the tumour. A catheter will then be placed in the afferent vessel that vascularises the tumour, and 150 to 180 MBq of ^99m^Tc-marked human serum albumin will be injected. The injection rate and catheter position will mimic the anticipated RE procedure. After the injection, the patient will have the pulmonary shunt evaluated using a dual-head gamma camera or single-photon emission computed tomography (SPECT)/computed tomography (CT). The prescribed activity of SIR-Spheres™ will then be calculated based on the patient’s body surface area and the percentage tumour involvement as described by Kennedy *et al*.[19]. Patients who would receive a dose in the lungs higher than 25 Gy based on the liver-to-lung shunt pre-assessment will not receive RE, but will remain in this study arm as part of the intention-to-treat group (ITT). Patients who are eligible for RE will commence treatment between the second and fifth week after randomisation with a single session of treatment (the day on which RE is administered is defined as D0 in Table 2). In patients with bilobar involvement, contralateral RE will be administered within 30 to 60 days. In order to avoid premature retreatment with RE of any lobe due to late tumour response, retreatment with RE will only be considered beyond 3 months from D0 in the absence of objective response or if there is significant progression on imaging (stable or progressor according to the response evaluation criteria in solid tumours (RECIST) or European Association for the Study of the Liver (EASL)) in the treated region (same tumour or new tumour). Retreatment with RE will also be considered beyond 6 weeks in the event of partial failure of the initial treatment due to an identified correctible cause or if an insufficient tumour dose was delivered.

### Trial assessments

The last enrolled patient will be followed up for 12 months after the start of treatment (D0). All other patients will be followed up until the final visit of the last enrolled patient. Patients will therefore be followed up for a maximum of 48 months and a minimum of 12 months following start of treatment. All patients will be assessed by the schedule summarised in Table 2. Treatment will be discontinued if the patient withdraws consent, if the treating physician deems it necessary for medical reasons or if a serious adverse event occurs - after discontinuation, patients should be assessed by CT as soon as possible to assess response to treatment.

### Outcome measures

The primary endpoint of the SARAH trial is OS. Secondary endpoints include: adverse events rate, progression-free survival (PFS) at 6 months according to RECIST[20], modified RECIST, EASL and Choi criteria; tumour response rate; general or liver disease-specific QoL scores; and cost of each strategy.

Cost endpoints are: the cost of RE from the hospital perspective; the average cost per patient from the payer’s perspective; and the incremental cost-effectiveness or cost-utility ratio.

### Outcome definitions

-  OS is defined as the time from the date of randomisation to death from any cause.
-  PFS - the time from the date of first treatment to disease progression - and tumour response rate (complete response, partial response, stability, or progression) will be determined from serial CT scans using RECIST, modified RECIST, EASL criteria for HCC, and Choi criteria[21]. Radiological examinations will be conducted by abdominal radiologists at each centre followed by a separate centralised review of radiological examinations.
-  The general and liver disease-specific quality of life scores will be calculated using the European Organisation for Research and Treatment of Cancer (EORTC) quality of life questionnaire (QLQ-C30) version 3 and the HCC-specific QLQ-HCC 18 questionnaire[22].
-  Adverse events will be reported according to National Cancer Institute criteria (National Cancer Institute Common Terminology criteria for Adverse Events (NCI CTCAE) Version 4.0)[23].

The cost of therapy from the hospital’s perspective will be estimated by including all the resources that are directly attributable to the procedure (that is, equipment, tests, total work time, and so on), which will be assigned a value based on the purchase price by the hospital. The mean overall cost per patient from the payer’s perspective will include the relative stay index and readmissions during the patient’s follow-up period. Calculation of the incremental cost-effectiveness ratio per year of survival or the incremental cost-utility ratio between RE and sorafenib will be complemented by the bootstrap resampling method and an acceptability curve for the cost-effectiveness ratio.

### Sample size calculation and statistical considerations

Based on OS data with sorafenib from the SHARP study[6] and with yttrium-90 RE reported in the literature[9, 11–15], the number of patients required for randomisation to detect a clinically relevant increase (4 months) in OS time with RE versus sorafenib was determined as 400 patients (200 patients in each treatment arm). This translates to an expected median OS time of 10.7 months in the sorafenib group and 15 months in the RE group, with an accrual period of 24 months and follow-up of 12 months. These guarantee a power of 80% with a bilateral alpha risk of 5%. Estimating that up to 10% of patients that are recruited will not fulfil the criteria of eligibility for randomisation, we aimed to enrol 440 patients.

A Data Monitoring Committee (DMC) will regularly review the toxicity data to assess the safety profile of the treatment (including serious adverse events and mortality). The first intermediate analysis will take place once 30 patients have been followed up for at least 2 months in each treatment arm, after which the DMC will convene every 6 months.

### Statistical analysis

Results will be reported according to the Consolidated Standards of Reporting Trials (CONSORT) statement. An ITT analysis will be performed, keeping patients in their randomisation group and including protocol deviations. A ‘per-protocol’ sensitivity analysis will also be performed. A study flowchart will be provided, including the number of patients who: are eligible; are randomised to receive treatment; are followed up; withdraw from the study; and are lost to follow-up. Major protocol deviations and the reasons for withdrawal from the study will be described.

The Kaplan and Meier method will be used to calculate survival (OS and PFS). The comparison of survival rates at 12 months between the two treatment groups will be performed using the log rank test (Mantel-Haenszel version). In addition, the treatment effect, once adjusted for the stratified randomisation factors, will be calculated via: (1) a stratified log rank analysis; and (2) a Cox’s regression model.

The median survival times (OS and PFS) in both treatment groups will be calculated, along with the confidence interval associated with the difference or with the median survival time ratio[24].

Toxicity will be reported according to NCI CTCAE Version 3.0, with particular reference to the proportion of patients experiencing grade 3/4 toxicity in each treatment arm.

The objective response will be determined via the RECIST and modified (m)RECIST criteria, the EASL criteria for HCC, and the Choi criteria, and a comparison will be made between the two treatment groups using the ‘best response during follow-up’ criterion. The response rates will be calculated by comparing the number of patients who responded during follow-up (complete or partial response) with the total number of randomised patients in each group. The related confidence intervals will be calculated and compared between the two groups using Pearson’s chi-squared test.

### Economic evaluation and statistical methods

The costs of both therapies will be compared using the Student’s *t* test. Uncertainty over the cost and effectiveness differentials between the two groups will be measured using the bootstrap resampling method. Cost-effectiveness will be measured using the incremental cost-effectiveness ratio per life year gained. Markov modelling will be used to calculate the cost-effectiveness ratio by simulating patient follow-up beyond the end of the study.

## Discussion

The SARAH trial will compare the efficacy and safety of RE with that of sorafenib in the treatment of advanced HCC (see Additional file2). To the authors’ knowledge, no prospective, controlled trials have been published, randomised or otherwise, on the efficacy of yttrium-90 RE in patients with HCC.

Sorafenib has been chosen as the control in the SARAH trial as it is the current recommended first-line treatment for advanced (Barcelona stage C) HCC[2, 6, 25]. Median OS is significantly increased by nearly 3 months with sorafenib versus placebo[6, 7]. However, the incidence of adverse events was high (80%) in the SHARP trial and there was no improvement in time to symptomatic progression with sorafenib therapy[6]. Attempts to improve outcomes for patients with advanced HCC on sorafenib by combination with other drug therapies have had limited success when compared with sorafenib alone[26–30]. Sorafenib combination therapy with transarterial chemoembolisation (TACE) has shown promise in patients with unresectable HCC[31], but is not currently recommended for advanced HCC. Indeed, combination therapy with sorafenib is not currently recommended outside the clinical trial setting for advanced HCC[27], and there is a need for an efficacious alternative with a favourable safety profile. RE with yttrium-90 is also indicated as a first-line treatment for unresectable HCC in a number of countries, and is associated with a high response rate in HCC patients (78 to 89% across 14 studies)[8]. Thus a head-to-head, prospective trial of these two treatments warrants investigation.

Some aspects of the SARAH study design are worth further discussion. The inclusion criteria are similar to the SHARP trial, except extrahepatic dissemination is not permitted in the SARAH study as RE is a localised therapy. Although previous treatment for advanced HCC is an exclusion criterion, prior chemoembolisation is permitted as patients failing chemoembolisation would be indicated for sorafenib therapy. Eligible patients in the SARAH study have also been stratified according to ECOG score, as this is an independent prognostic factor for survival in patients with HCC treated with RE or sorafenib[10, 32]. In addition, patients will be stratified by presence or absence of macroscopic vascular involvement as vascular involvement has been associated with poor prognosis in patients with HCC[10, 13, 33].

In the SARAH trial, OS has been chosen as the primary endpoint as it is a more robust measure than PFS, and the SHARP trial demonstrated the efficacy of sorafenib based on this criterion. Moreover, the kinetics of tumour progression, as assessed from imaging techniques, is different between RE and sorafenib, rendering OS the best option for comparison between arms in this trial. An Asian study with similar inclusion criteria has now commenced, which compares sorafenib with RE in patients with locally advanced HCC[34], and could be used for meta-analysis in the future.

In addition to efficacy analyses, the SARAH trial offers the possibility of rigorously confirming the toxicity caused by sorafenib versus RE in patients with advanced HCC. This is important as the cardiovascular toxicity of sorafenib has been highlighted in a meta-analysis of cancer patients (predominantly renal carcinoma)[35], but was not a common complication in the SHARP study[6]. The SARAH trial will also offer the opportunity to conduct ancillary studies (for example, dosimetry and CT perfusion), and compare the cost of each treatment. A definitive economic evaluation of sorafenib therapy versus RE for advanced HCC is not currently available. It is therefore useful to: conduct a study using national data to establish the value of the resources used; take the specificities of local oncology practices into account; and compare sorafenib treatment to an up-to-date therapy, RE.

Limitations to the SARAH study design have been combated where feasible. While blinding is not possible due to the treatment methods, the potential biases caused by the lack of blinding have been minimised by the choice of OS as a robust primary endpoint. In addition, it is planned that an independent group of radiologists will perform a blind review of the imaging in order to guarantee the absence of bias regarding PFS.

The results from the SARAH trial should further the understanding of RE and determine the optimal treatment modality in advanced HCC. In addition, the data generated from this study may help to place RE into future consensus guidelines.

## Trial status

The SARAH trial is currently recruiting participants.

## Electronic supplementary material

Additional file 1: Approval of ethics committee for all participating centres.(PDF 191 KB)

Additional file 2: Brief summary.(DOCX 45 KB)

Below are the links to the authors’ original submitted files for images.

Authors’ original file for figure 1

## Abbreviations

^99m^Tc: technetium-99 m
AASLD: American Association for the Study of Liver Diseases
ALT: alanine aminotransferase
AST: aspartate transaminase
CONSORT: Consolidated Standards of Reporting Trials
CT: computed tomography
DMC: Data Monitoring Committee
EASL: European Association for the Study of the Liver
ECOG: Eastern Cooperative Oncology Group
EORTC: European Organisation for Research and Treatment of Cancer
HCC: hepatocellular carcinoma
INR: international normalised ratio
ITT: intention to treat
NCI CTCAE: National Cancer Institute Common Terminology Criteria for Adverse Events
OS: overall survival
PFS: progression-free survival
QoL: quality of life
RE: radioembolisation
RECIST: response evaluation criteria in solid tumours
SARAH: SorAfenib versus Radioembolisation in Advanced Hepatocellular carcinoma
SHARP: Sorafenib Hepatocellular carcinoma Assessment Randomized Protocol
SIRT: selective internal radiation therapy
SPECT: single-photon emission computed tomography
TACE: transarterial chemoembolisation
ULN: upper limit of normal.

## References

[1] Venook AP, Papandreou C, Furuse J, de Guevara LL (2010). The incidence and epidemiology of hepatocellular carcinoma: a global and regional perspective. Oncologist.

[2] European Association for the Study of the Liver, European Organisation for Research and Treatment of Cancer (2012). EASL–EORTC clinical practice guidelines: management of hepatocellular carcinoma. J Hepatol.

[3] Cabbibo G, Enea M, Latteri F, Genco C, Craxì A, Cammá C (2009). Survival of unresectable hepatocellular carcinoma: a meta-analysis of the control arms of 28 randomized trials. J Hepatol.

[4] Hsu C, Shen YC, Cheng CC, Hu FC, Cheng AL (2010). Geographic difference in survival outcome for advanced hepatocellular carcinoma: implications on future clinical trial design. Contemporary Clinical Trial.

[5] Llovet JM, Di Bisceglie AM, Bruix J, Kramer BS, Lencioni R, Zhu AX, Sherman M, Schwartz M, Lotze M, Talwalkar J, Gores GJ (2008). Design and endpoints of clinical trials in hepatocellular carcinoma. J Natl Cancer Inst.

[6] Llovet JM, Ricci S, Mazzaferro V, Hilgard P, Gane E, Blanc JF, de Oliveira AC, Santoro A, Raoul JL, Forner A, Schwartz M, Porta C, Zeuzem S, Bolondi L, Greten TF, Galle PR, Seitz JF, Borbath I, Häussinger D, Giannaris T, Shan M, Moscovici M, Voliotis D, Bruix J, SHARP Investigators Study Group (2008). Sorafenib in advanced hepatocellular carcinoma. N Engl J Med.

[7] Cheng AL, Kang YK, Chen Z, Tsao CJ, Qin S, Kim JS, Luo R, Feng J, Ye S, Yang TS, Xu J, Sun Y, Liang H, Liu J, Wang J, Tak WY, Pan H, Burock K, Zou J, Voliotis D, Guan Z (2009). Efficacy and safety of sorafenib in patients in the Asia-Pacific region with advanced hepatocellular carcinoma: a phase III randomised, double-blind, placebo-controlled trial. Lancet Oncol.

[8] Vente MA, Wondergem M, van der Tweel I, van den Bosch MA, Zonnenberg BA, Lam MG, van Het Schip AD, Nijsen JF (2009). Yttrium-90 microsphere radioembolization for the treatment of liver malignancies: a structured meta-analysis. Eur Radiol.

[9] Kulik LM, Atassi B, van Holsbeeck L, Souman T, Lewandowski RJ, Mulcahy MF, Hunter RD, Nemcek AA, Abecassis MM, Haines KG, Salem R (2006). Yttrium-90 microspheres (TheraSphere) treatment of unresectable hepatocellular carcinoma: downstaging to resection, RFA and bridge to transplantation. J Surg Oncol.

[10] Sangro B, Carpanese L, Cianni R, Golfieri R, Gasparini D, Ezziddin S, Paprottka PM, Fiore F, Van Buskirk M, Bilbao JI, Ettorre GM, Salvatori R, Giampalma E, Geatti O, Wilhelm K, Hoffmann RT, Izzo F, Iñarrairaegui M, Maini CL, Urigo C, Cappelli A, Vit A, Ahmadzadehfar H, Jakobs TF, Lastoria S, European Network on Radioembolization with Yttrium-90 Resin Microspheres (ENRY) (2011). Survival after yttrium-90 resin microsphere radioembolization of hepatocellular carcinoma across Barcelona clinic liver cancer stages: a European evaluation. Hepatology (Baltimore, MD).

[11] Lau WY, Ho S, Leung TW, Chan M, Ho R, Johnson PJ, Li AK (1998). Selective internal radiation therapy for nonresectable hepatocellular carcinoma with intraarterial infusion of 90yttrium microspheres. Int J Radiat Oncol Biol Phys.

[12] Lau WY, Leung WT, Ho S, Leung NW, Chan M, Lin J, Metreweli C, Johnson P, Li AK (1994). Treatment of inoperable hepatocellular carcinoma with intrahepatic arterial yttrium-90 microspheres: a phase I and II study. Br J Cancer.

[13] Kulik LM, Carr BI, Mulcahy MF, Lewandowski RJ, Atassi B, Ryu RK, Sato KT, Benson A, Nemcek AA, Gates VL (2008). Safety and efficacy of 90Y radiotherapy for hepatocellular carcinoma with and without portal vein thrombosis. Hepatology (Baltimore, MD).

[14] Sangro B, Bilbao JI, Boan J, Martinez-Cuesta A, Benito A, Rodriguez J, Panizo A, Gil B, Inarrairaegui M, Herrero I, Quiroga J, Prieto J (2006). Radioembolization using 90Y-resin microspheres for patients with advanced hepatocellular carcinoma. Int J Radiat Oncol Biol Phys.

[15] Young JY, Rhee TK, Atassi B, Gates VL, Kulik L, Mulcahy MF, Larson AC, Ryu RK, Sato KT, Lewandowski RJ, Omary RA, Salem R (2007). Radiation dose limits and liver toxicities resulting from multiple yttrium-90 radioembolization treatments for hepatocellular carcinoma. J Vasc Intervent Radiol.

[16] Hilgard P, Hamami M, El Fouly A, Scherag A, Müller S, Ertle J, Heusner T, Cicinnati VR, Paul A, Bockisch A, Gerken G, Antoch G (2010). Radioembolization with yttrium-90 glass microspheres in hepatocellular carcinoma: European Experience on safety and long-term survival. Hepatology.

[17] Mazzaferro V, Sposito C, Bhoori S, Romito R, Chiesa C, Morosi C, Maccauro M, Marchianò A, Bongini M, Lanocita R, Civelli E, Bombardieri E, Camerini T, Spreafico C (2013). Yttrium-90 radioembolization for intermediate-advanced hepatocellular carcinoma: a phase 2 study. Hepatology.

[18] Salem R, Lewandowski RJ, Mulcahy MF, Riaz A, Ryu RK, Ibrahim S, Atassi B, Baker T, Gates V, Miller FH, Sato KT, Wang E, Gupta R, Benson AB, Newman SB, Omary RA, Abecassis M, Kulik L (2010). Radioembolization for hepatocellular carcinoma using yttrium-90 microspheres: a comprehensive report of long-term outcomes. Gastroenterology.

[19] Kennedy AS, Kleinstreuer C, Basciano CA, Dezarn WA (2010). Computer modeling of yttrium-90-microsphere transport in the hepatic arterial tree to improve clinical outcomes. Int J Radiat Oncol Biol Phys.

[20] Therasse P, Arbuck SG, Eisenhauer EA, Wanders J, Kaplan RS, Rubinstein L, Verweij J, Van Glabbeke M, van Oosterom AT, Christian MC, Gwyther SG (2000). New guidelines to evaluate the response to treatment in solid tumors. European Organization for Research and Treatment of Cancer, National Cancer Institute of the United States, National Cancer Institute of Canada. J Natl Cancer Inst.

[21] Ronot M, Bouattour M, Wassermann J, Bruno O, Dreyer C, Larroque B, Castera L, Vilgrain V, Belghiti J, Raymond E, Faivre S (2014). Alternative Response Criteria (Choi, European association for the study of the liver, and modified Response Evaluation Criteria in Solid Tumors [RECIST]) Versus RECIST 1.1 in patients with advanced hepatocellular carcinoma treated with sorafenib. Oncologist.

[22] Blazeby JM, Currie E, Zee BC, Chie WC, Poon RT, Garden OJ, Group EQoL (2004). Development of a questionnaire module to supplement the EORTC QLQ-C30 to assess quality of life in patients with hepatocellular carcinoma, the EORTC QLQ-HCC18. Eur J Cancer.

[23] Common Terminology Criteria for Adverse Events (CTCAE) Version 4.0. http://evs.nci.nih.gov/ftp1/CTCAE/CTCAE_4.03_2010-06-14_QuickReference_5x7.pdf]

[24] Su JQ, Wei LJ (1993). Nonparametric estimation for the difference or ratio of median failure times. Biometrics.

[25] El-Serag HB, Marrero JA, Rudolph L, Reddy KR (2008). Diagnosis and treatment of hepatocellular carcinoma. Gastroenterology.

[26] Abou-Alfa GK, Johnson P, Knox JJ, Capanu M, Davidenko I, Lacava J, Leung T, Gansukh B, Saltz LB (2010). Doxorubicin plus sorafenib vs doxorubicin alone in patients with advanced hepatocellular carcinoma: a randomized trial. JAMA.

[27] Abdel-Rahman O, Fouad M (2014). Sorafenib-based combination as a first line treatment for advanced hepatocellular carcinoma: a systematic review of the literature. Crit Rev Oncol/Hematol.

[28] Gomez-Martin C, Bustamante J, Castroagudin JF, Salcedo M, Garralda E, Testillano M, Herrero I, Matilla A, Sangro B (2012). Efficacy and safety of sorafenib in combination with mammalian target of rapamycin inhibitors for recurrent hepatocellular carcinoma after liver transplantation. Liver Transplant.

[29] Petrini I, Lencioni M, Ricasoli M, Iannopollo M, Orlandini C, Oliveri F, Bartolozzi C, Ricci S (2012). Phase II trial of sorafenib in combination with 5-fluorouracil infusion in advanced hepatocellular carcinoma. Cancer Chemother Pharmacol.

[30] Prete SD, Montella L, Caraglia M, Maiorino L, Cennamo G, Montesarchio V, Piai G, Febbraro A, Tarantino L, Capasso E, Palmieri G, Guarrasi R, Bianco M, Mamone R, Savastano C, Pisano A, Vincenzi B, Sabia A, D'Agostino A, Faiola V, Addeo R (2010). Sorafenib plus octreotide is an effective and safe treatment in advanced hepatocellular carcinoma: multicenter phase II So.LAR. study. Cancer Chemother Pharmacol.

[31] Pawlik TM, Reyes DK, Cosgrove D, Kamel IR, Bhagat N, Geschwind JF (2011). Phase II trial of sorafenib combined with concurrent transarterial chemoembolization with drug-eluting beads for hepatocellular carcinoma. J Clin Oncol.

[32] Wang C, Lu Y, Wang H, Gao X, Bai W, Qu J, Xu G, Zhang Z, Zeng Z, Zhou L, An L, Lv J, Yang Y (2012). Transarterial chemoembolization with/without cryotherapy is associated with improved clinical outcomes of sorafenib for the treatment of advanced hepatocellular carcinoma. Exp Therapeut Med.

[33] Kudo M, Ueshima K, Arizumi T (2012). Real-life clinical practice with sorafenib in advanced hepatocellular carcinoma: a single-center experience. Dig Dis.

[34] NCT01135056. Study to compare selective internal radiation therapy (SIRT) versus sorafenib in locally advanced hepatocellular carcinoma (HCC). [http://clinicaltrials.gov/ct2/show/NCT01135056?term=sorafenib+hepatocellular+carcinoma+yttrium-90%26rank=5],

[35] Wu S, Chen JJ, Kudelka A, Lu J, Zhu X (2008). Incidence and risk of hypertension with sorafenib in patients with cancer: a systematic review and meta-analysis. Lancet Oncol.

