# Supplementary material for: Radioembolisation with yttrium‒90 microspheres versus sorafenib for treatment of advanced hepatocellular carcinoma (SARAH): study protocol for a randomised controlled trial
Source: Trials. 2014 Dec 3;15:474. doi: 10.1186/1745-6215-15-474 (PMC4265525; doi:10.1186/1745-6215-15-474)
Supplement: Supplementary file 1 — Additional file 1: Approval of ethics committee for all participating centres.(PDF 191 KB) [file 13063_2014_2333_MOESM1_ESM.pdf]

The following ethics committee approved the SARAH trial for all participating centres:

CPP Ile de France XI, 20, rue Armagis, 78105 Saint Germain en Laye Cedex

Reference number: 11 051

N° IDRCB (EUDRA CT number): 2011-A00043-38

**Participating centre**	**Town/city**	**Date ethics approval obtained**
Hôpital Beaujon	Clichy	09/06/2011
CHU d'Amiens	Amiens	09/06/2011
CHU d'Angers	Angers	09/06/2011
CHU Hôpital Côte de Nacre	Caen	09/02/2012
CHU de Grenoble	Grenoble	09/06/2011
CHU Edouard Herriot	Lyon	09/06/2011
CCLC Paoli-Calmette	Marseille	09/06/2011
Hôpital Saint Eloi	Montpellier	09/06/2011
Hôpital de Brabois (CHU Nancy)	Vandoeuvre les Nancy	09/06/2011
Hôpital Hôtel Dieu	Nantes	09/06/2011
CHU de Nice (Archet 1 et 2)	Nice	09/02/2012
Hôpital Saint Antoine	Paris Cedex 12	09/06/2011
HEGP	Paris Cedex 15	09/06/2011
Hôpital Haut Lévèque	Pessac	09/06/2011
CHU de Poitiers	Poitiers	09/06/2011
Hôpital Nord - CHU de Saint Etienne	Saint Priest en Jarez	09/06/2011
Hôpital de Hautepierre	Strasbourg	09/06/2011
Nouvel Hôpital Civil	Strasbourg	13/06/2013
Paul Brousse	Villejuif	09/06/2011
IGR	Villejuif	09/02/2012
CHRU de Besançon, Hôpital Jean Minjoz	Besançon	09/02/2012
Bocage	Dijon	09/02/2012
Centre Georges François LECLERC	Dijon	11/10/2012
CHU de Marseille, Hôpital la Timone	Marseille cedex 5	09/02/2012
CHU Henri Mondor	Créteil	11/10/2012
Hôpital Jean Verdier	Bondy	11/10/2012
Hôpital Lyon Sud	Pierre Bénite	13/06/2013
Hôpital de la croix rousse	Lyon Cedex 04	13/06/2013
CHU Robert DEBRE	Reims	13/06/2013
Hôpital Antoine Béclère	Clamart	13/06/2013
Hôpital Le Kremlin Bicêtre	Kremlin-Bicêtre Cedex	13/06/2013
