# Supplementary material for: Radioembolisation with yttrium‒90 microspheres versus sorafenib for treatment of advanced hepatocellular carcinoma (SARAH): study protocol for a randomised controlled trial
Source: Trials. 2014 Dec 3;15:474. doi: 10.1186/1745-6215-15-474 (PMC4265525; doi:10.1186/1745-6215-15-474)
Supplement: Supplementary file 2 — Additional file 2: Brief summary.(DOCX 45 KB) [file 13063_2014_2333_MOESM2_ESM.docx]

**Radioembolisation with yttrium‐90 microspheres versus sorafenib for treatment of advanced hepatocellular carcinoma (SARAH): study protocol for a randomized controlled trial.**

The SARAH trial has been designed to compare the efficacy and safety of sorafenib therapy and Radioembolisation using yttrium-90 resin microspheres in patients with advanced HCC. The primary endpoint of the SARAH trial is overall survival. Quality of life (QoL) and cost-effectiveness will also be compared between therapies.
