# Supplementary figures and images for: Radioembolisation with yttrium‒90 microspheres versus sorafenib for treatment of advanced hepatocellular carcinoma (SARAH): study protocol for a randomised controlled trial
Source: Trials. 2014 Dec 3;15:474. doi: 10.1186/1745-6215-15-474 (PMC4265525; doi:10.1186/1745-6215-15-474)

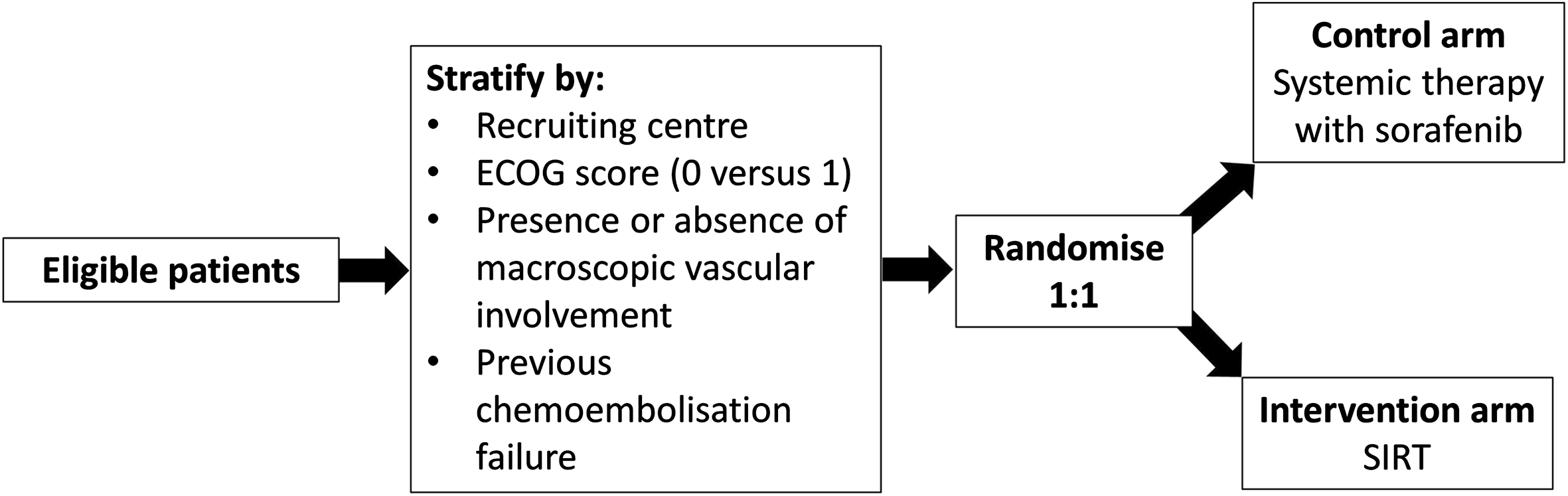

Supplement: Supplementary file 3 — Authors’ original file for figure 1 [file 13063_2014_2333_MOESM3_ESM.tif]
